# High-dimensional detection of imaging response to treatment in multiple sclerosis

**DOI:** 10.1038/s41746-019-0127-8

**Published:** 2019-06-10

**Authors:** Baris Kanber, Parashkev Nachev, Frederik Barkhof, Alberto Calvi, Jorge Cardoso, Rosa Cortese, Ferran Prados, Carole H. Sudre, Carmen Tur, Sebastien Ourselin, Olga Ciccarelli

**Affiliations:** 1 Centre for Medical Image Computing, Department of Medical Physics and Biomedical Engineering, University College London, London, UK; 2 Multiple Sclerosis Research Centre, NMR Research Unit, Department of Neuroinflammation, Queen Square Institute of Neurology, University College London, London, UK; 3 National Institute for Health Research (NIHR) University College London Hospitals Biomedical Research Centre (BRC), London, UK; 4 High Dimensional Neurology Group, Queen Square Institute of Neurology, University College London, London, UK; 5 Department of Radiology & Nuclear Medicine, VU University Medical Center, Amsterdam, The Netherlands; 6 School of Biomedical Engineering & Imaging Sciences, King’s College London, London, UK

**Keywords:** Multiple sclerosis, Translational research

## Abstract

Changes on brain imaging may precede clinical manifestations or disclose disease progression opaque to conventional clinical measures. Where, as in multiple sclerosis, the pathological process has a complex anatomical distribution, such changes are not easily detected by low-dimensional models in common use. This hinders our ability to detect treatment effects, both in the management of individual patients and in interventional trials. Here we compared the ability of conventional models to detect an imaging response to treatment against high-dimensional models incorporating a wide multiplicity of imaging factors. We used fully-automated image analysis to extract 144 regional, longitudinal trajectories of pre- and post- treatment changes in brain volume and disconnection in a cohort of 124 natalizumab-treated patients. Low- and high-dimensional models of the relationship between treatment and the trajectories of change were built and evaluated with machine learning, quantifying performance with receiver operating characteristic curves. Simulations of randomised controlled trials enrolling varying numbers of patients were used to quantify the impact of dimensionality on statistical efficiency. Compared to existing methods, high-dimensional models were superior in treatment response detection (area under the receiver operating characteristic curve = 0.890 [95% CI = 0.885–0.895] vs. 0.686 [95% CI = 0.679–0.693], *P* < 0.01]) and in statistical efficiency (achieved statistical power = 0.806 [95% CI = 0.698–0.872] vs. 0.508 [95% CI = 0.403–0.593] with number of patients enrolled = 50, at *α* = 0.01). High-dimensional models based on routine, clinical imaging can substantially enhance the detection of the imaging response to treatment in multiple sclerosis, potentially enabling more accurate individual prediction and greater statistical efficiency of randomised controlled trials.

## Introduction

The value of any therapy is ultimately determined by its impact on patients’ lives. Both clinical and investigational measures are merely surrogates of impact, the former favoured owing to its perceived directness. Nonetheless, under two circumstances the imaging response to an intervention may be more important than the clinical. First, when imaging changes precede clinical manifestations they can provide grounds for earlier intervention. Oncologists, for example, rarely wait for an imaging relapse to become clinically obvious. Second, when the impact on quality-of-life is difficult to quantify, an imaging response may obtain greater real-world accuracy. In focal brain injury, for example, though the behavioural effects of prefrontal damage obvious on imaging can be devastating, even sophisticated neuropsychological testing often fails to detect them.^1^

Both these circumstances apply to multiple sclerosis. Imaging changes precede clinical deterioration, support the diagnosis and predict the prognosis of multiple sclerosis,^2^ and are surrogates for relapses.^3^ Clinical measures neither capture all affected functional domains nor the progression of disability.^4^ Furthermore, a divide exists between detecting an individual response in routine clinical care, and detecting an “average” response within clinical trials.

A means of detecting an imaging response to treatment is therefore needed: the question is how best to achieve it. The conventional approach identifies a small set of univariate “biomarkers”, such as the number of lesions and volume, indexing their response to therapy within univariate or low-dimensional, multivariate statistical models. But brain pathology here extends beyond visible lesions, involves grey and white matter, and interacts with the complexity of the brain’s functional anatomy in a way that only a large number of features could adequately characterise.^5^ Indeed, multivariate patterns of grey matter atrophy are both complex and clinically relevant.^6^ Low-dimensional models that ignore this complexity are therefore bound to be insensitive to treatment effects.

Until recently, the difficulty of extracting multiple imaging features and evaluating them within high-dimensional multivariate models has limited the application of complex modelling to this problem. But automated tissue segmentation and anatomical parcellation can now quantify regional brain atrophy at high spatial resolution,^7^ and white matter lesion segmentation, coupled with tractography, can define complex patterns of grey matter disconnection, yielding a high-dimensional, multivariate “fingerprint” of the brain. Conventional, low-dimensional models can now be compared with the proposed, high-dimensional models, quantifying the impact on the sensitivity and specificity for detecting a treatment response.^8–12^

Crucially, this approach is applicable to real-world, heterogeneous magnetic resonance imaging (MRI) data. Where the biological signal is high-dimensional, conveyed in the interactions between many features, it becomes resistant to noise and baseline variability, given sufficient data and the right modelling technique. The ability to use real-world, heterogeneous MRI data would make it possible for high-dimensional modelling to be readily introduced into clinical practice and interventional clinical trials.

Recent publications have shown the value of high-dimensional modelling based on brain lesion patterns in predicting conversion to multiple sclerosis,^9^ future disease activity^10^ and treatment responders,^11^ establishing the theoretical basis for the work described in this paper. Given that brain morphology is also important in the prediction of clinical outcomes,^12^ one can hypothesise that the addition of the latter would benefit the detection of imaging response to treatment, and we test this hypothesis.

Here we examined an unselected cohort of relapsing-remitting patients with multiple sclerosis treated with natalizumab, and imaged with standard-of-care MRI. Our aim was to compare the performance of conventional low- and the proposed, high-dimensional models in detecting the imaging response to the treatment in the real-world clinical setting, and by simulating a randomised, clinical trial. We further sought to define the potential applications of such models.

## Results

### Performance figures

**Fig. 1:**
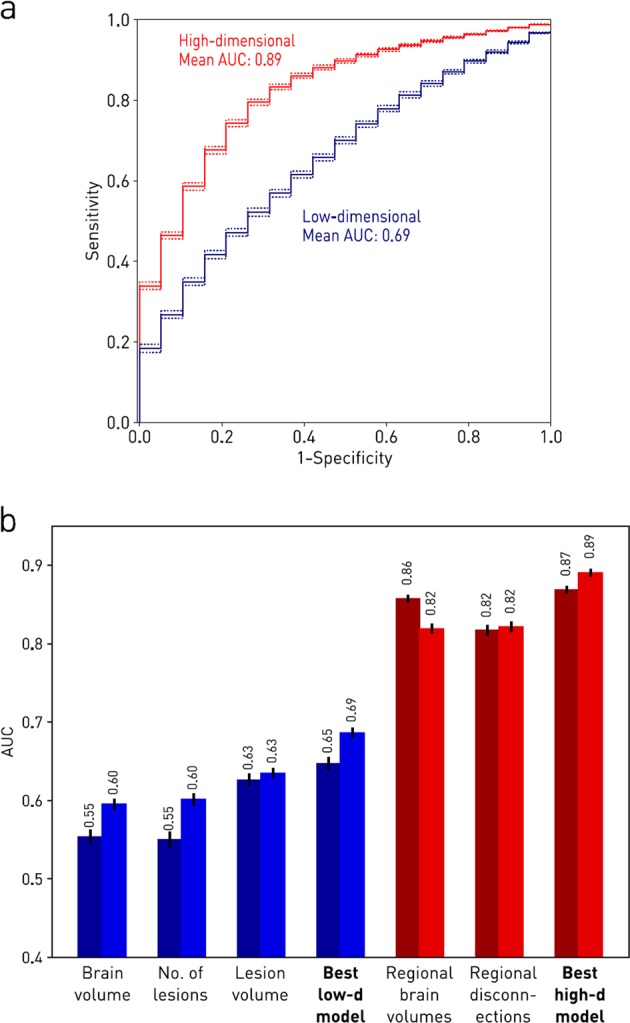
Imaging response detection with low- and high-dimensional models. **a** Receiver operating characteristic curves for imaging response detection using the best high- and low-dimensional models. Solid lines indicate the mean performance of each model, and dashed lines the 95% confidence intervals for the mean. **b** Imaging response detection performance for each model and the two types of classifier obtained with two different machine learning techniques. Bars indicate the mean performance of each model and the lines the 95% confidence intervals for the mean. Low-dimensional models are drawn in blue, while the high-dimensional models are drawn in red. Darker colour bars indicate the support vector machine classifiers; the lighter colour bars indicate the extremely randomised trees classifiers. All high-dimensional models are seen to outperform the best low-dimensional model, regardless of the classifier used

The best high-dimensional model (ERT classifier) yielded a significantly superior performance than the best, conventional, low-dimensional model (ERT classifier): high-dimensional ERT model AUC = 0.890 (95% confidence intervals (CIs): 0.885–0.895) vs. low-dimensional ERT model AUC = 0.686 (95% CIs: 0.679–0.693) (*P* < 0.01) (Fig. 1a). This reflected an individual accuracy of 86.5% (95% CIs: 85.9–87.0), a sensitivity of 77.7% (95% CIs: 77.2–78.3) and a specificity of 77.8% (95% CIs: 77.3–78.2) for the best high-dimensional model (ERT classifier), compared with an accuracy of 66.4% (95% CIs: 65.8–66.9), a sensitivity of 61.8% (95% CIs: 61.2–62.4) and a specificity of 66.8% (95% CIs: 66.2–67.5) for the best, conventional, low-dimensional model (ERT classifier). The superiority of the high-dimensional approach was consistent across both types of machine learning classifiers (Fig. 1b, Table 1). Similar results were obtained with data balanced by subsampling (see Supplementary Material). High-dimensional models that incorporated volume and disconnectome dimensions simultaneously achieved similar performance to those reported above (results not shown). Null models constructed by randomly permuting the order of values in each feature time series before computing slopes, and thus trajectories, could not reliably detect the intervention, confirming that the observed performance was not due to differences in scan timing (Table 2).

**Table 1:** Imaging response detection performance of each model and the two types of classifier

Predictor set	Support vector machines	Extremely randomised trees
Brain volume	0.554 [0.545–0.563]	0.595 [0.588–0.602]
Number of lesions	0.550 [0.540–0.561]	0.601 [0.594–0.609]
Total lesion volume	0.626 [0.618–0.634]	0.635 [0.629–0.641]
Best low dimensional	0.647 [0.639–0.655]	0.686 [0.679–0.693]
Regional atrophy	0.857 [0.852–0.862]	0.819 [0.813–0.825]
Regional disconnection	0.817 [0.810–0.824]	0.822 [0.815–0.828]
Best high dimensional	0.869 [0.864–0.873]	0.890 [0.885–0.895]

The best high-dimensional model was constructed as one which provided an average of the predictions made by the regional atrophy and the regional disconnection models, weighted by their corresponding mean AUCs. All figures are given as mean AUC [95% CI]

**Table 2:** Imaging response detection performance of the null models

Predictor set	Support vector machines	Extremely randomised trees
Brain volume	0.469 [0.463–0.475]	0.498 [0.490–0.506]
Number of lesions	0.457 [0.449–0.465]	0.464 [0.458–0.470]
Total lesion volume	0.473 [0.465–0.480]	0.535 [0.528–0.542]
Best low dimensional	0.497 [0.490–0.503]	0.473 [0.467–0.480]
Regional atrophy	0.445 [0.437–0.453]	0.454 [0.447–0.461]
Regional disconnection	0.465 [0.457–0.473]	0.467 [0.459–0.476]
Best high dimensional	0.459 [0.450–0.469]	0.422 [0.414–0.430]

None of the null models could reliably detect the intervention. All figures are given as mean AUC [95% CI]

### Imaging features

**Fig. 2:**
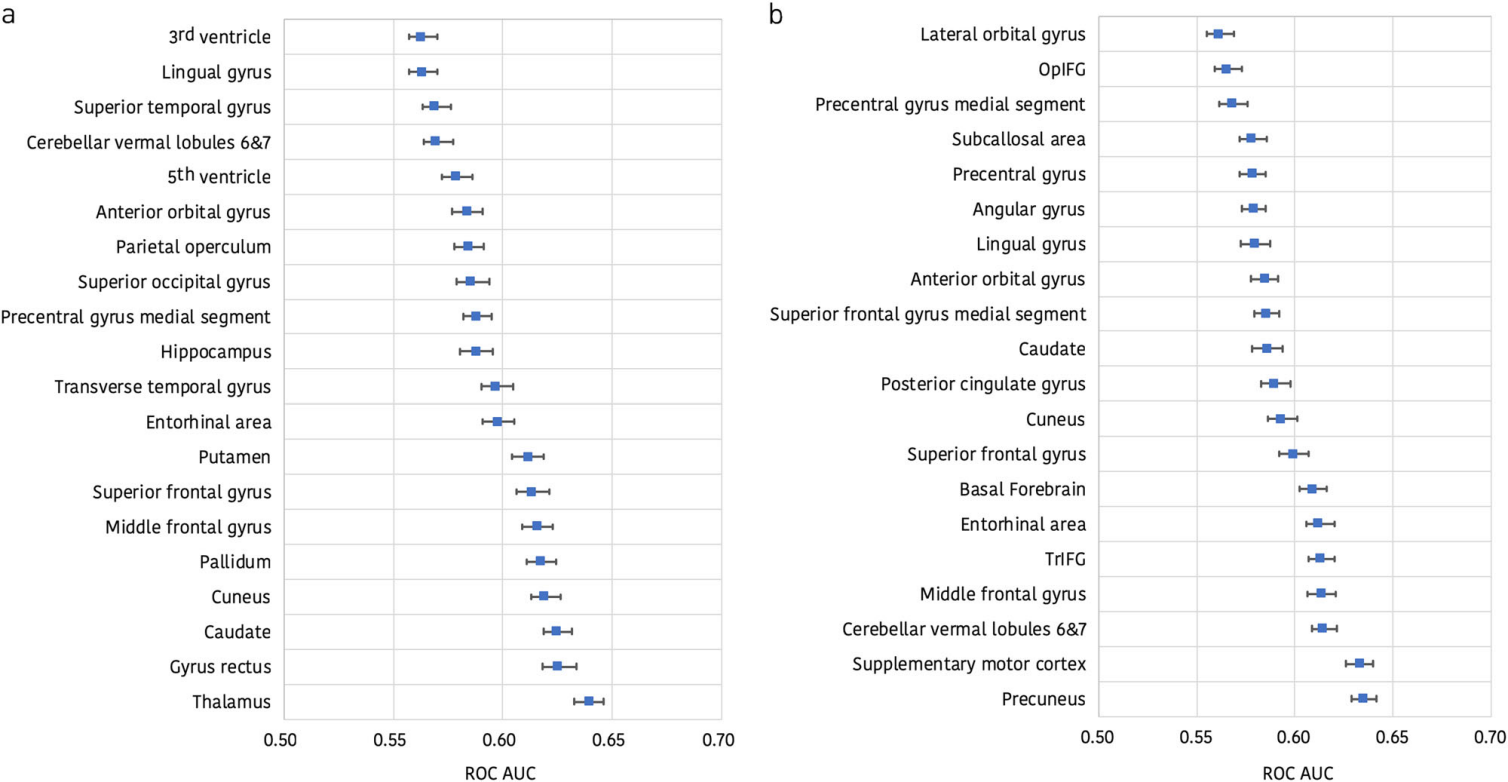
Regional brain volume and disconnectome trajectories most relevant to detecting the imaging response to treatment. The horizontal axes show the maximum of the two mean areas under the receiver operating characteristic curve with 95% confidence intervals obtained using the SVM and ERT classifiers when each regional brain volume (**a**) and each regional disconnectome (**b**) predictor is used in isolation. The following abbreviations have been used: OpIFG opercular part of the inferior frontal gyrus, TrIFG triangular part of the inferior frontal gyrus

**Fig. 3:**
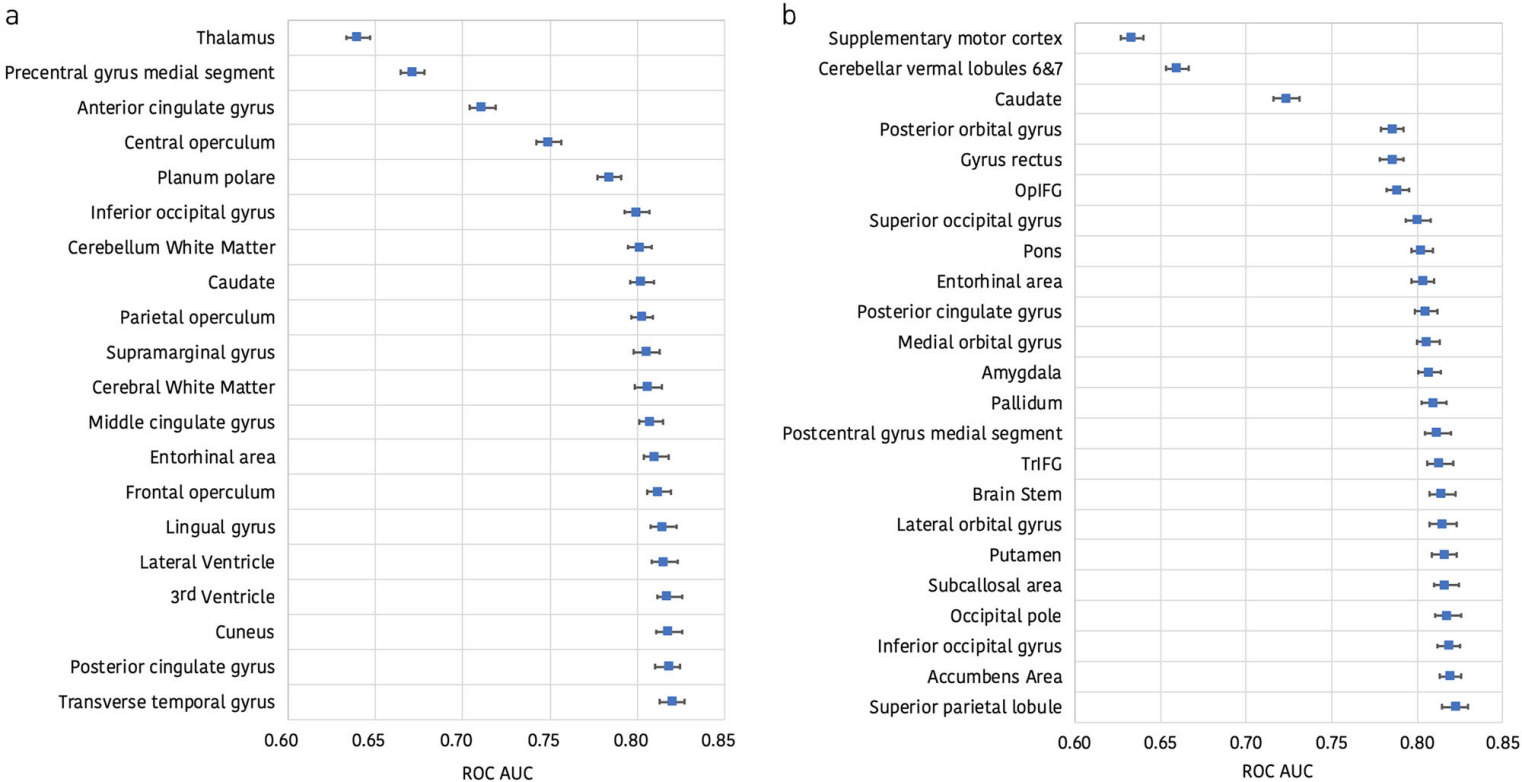
Brain regions included in the best high-dimensional ERT model based solely on regional brain volume and disconnectome trajectories. **a** The horizontal axis shows the mean area under the receiver operating characteristic (ROC) curve with 95% confidence intervals as each regional brain volume predictor is added to the model starting with the thalamus. **b** The horizontal axis shows the mean area under the ROC curve with 95% confidence intervals as each regional brain disconnectome predictor is added to the model starting with the supplementary motor cortex. OpIFG opercular part of the inferior frontal gyrus, TrIFG triangular part of the inferior frontal gyrus

**Fig. 4:**
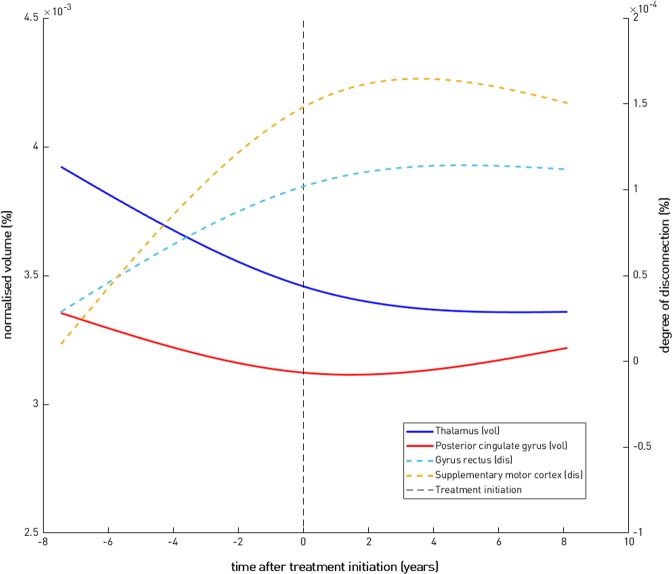
Direction of association for four representative brain regions. This figure shows that, at the population level, and for two representative regions, namely the thalamus (blue solid line) and the posterior cingulate gyrus (red solid line), we consistently observe atrophy (volume decrease) in the pre-treatment period (*t* < 0) and stabilisation/recovery post-treatment (*t* > 0). For the degree of disconnection, and for two other representative regions, namely the gyrus rectus (cyan dashed line) and the supplementary motor cortex (light brown dashed line), the direction of association is the opposite (but clinically the same), namely an increase in the degree of disconnection in the pre-treatment period and stabilisation/recovery post-treatment

The imaging features that were found to be most valuable for detecting the imaging response to treatment (Fig. 2) and those that comprised the best high-dimensional model based solely on regional brain volume trajectories and the best high-dimensional model based solely on regional brain volume disconnectome trajectories (Fig. 3) were consistent with known patterns of lesion and parenchymal change in multiple sclerosis. They were also concordant in the direction of their effects (Fig. 4).

### Confounders

The number of scans used for confounder regression and the number of scans used specifically for the detection of imaging response to treatment were 563 (124 patients) and 485 (103 patients), respectively. None of confounders could reliably be predicted using the confounder adjusted imaging-derived parameters, confirming the absence of residual covariate effects following confounder regression (Table 3).

**Table 3:** Verification of the absence of residual confounder effects following confounder regression

Confounder	Before confounder regression	After confounder regression
Age^a,b^	0.645 [0.629–0.661]	0.483 [0.472–0.494]
Gender	0.723 [0.711–0.736]	0.488 [0.475–0.501]
Scanner manufacturer^c^	0.768 [0.756–0.780]	0.503 [0.487–0.519]
Field strength^d^	0.803 [0.790–0.815]	0.503 [0.489–0.516]
Disease duration^a,b^	0.506 [0.493–0.519]	0.488 [0.477–0.499]
EDSS^a,b^	0.533 [0.511–0.555]	0.502 [0.483–0.521]
T1 slice thickness^e^	0.522 [0.488–0.556]	0.442 [0.408–0.477]
T1 voxel resolution^b^	0.789 [0.775–0.803]	0.497 [0.483–0.511]
FLAIR slice thickness^e^	0.627 [0.591–0.662]	0.498 [0.474–0.523]
FLAIR voxel resolution^b^	0.717 [0.699–0.736]	0.504 [0.491–0.517]

None of the confounders could reliably be predicted using the imaging data by a support vector machine classifier following confounder regression. All figures are given as mean AUC [95% confidence interval]

^a^At the start of treatment with natalizumab

^b^Predicting whether the confounder value is less than the mean for the sample population or otherwise

^c^The most common manufacturer in the sample population vs. the rest

^d^1.5 T vs. 3.0 T

^e^Predicting whether the slice thickness is less than 6 mm or otherwise

The class distribution was 33.1% class 0 (pre-treatment) and 66.9% class 1 (post-treatment), while the robust performance metrics of balanced *F*-scores and Matthews Correlation Coefficients also confirmed the superiority of the high-dimensional models (Tables 4 and 5).

**Table 4:** Additional imaging response detection performance metrics (balanced *F*-scores) for each model and the two types of classifier

Predictor set	Support vector machines	Extremely randomised trees
Brain volume	0.555 [0.541–0.569]	0.601 [0.593–0.609]
Number of lesions	0.595 [0.581–0.609]	0.666 [0.655–0.677]
Total lesion volume	0.602 [0.592–0.612]	0.619 [0.610–0.628]
Best low dimensional	0.722 [0.713–0.731]	0.717 [0.708–0.725]
Regional atrophy	0.849 [0.843–0.855]	0.835 [0.829–0.842]
Regional disconnection	0.821 [0.814–0.829]	0.816 [0.809–0.823]
Best high dimensional	0.876 [0.870–0.882]	0.874 [0.868–0.880]

All figures are given as the mean, balanced *F*-score [95% CI]

**Table 5:** Additional imaging response detection performance metrics (Matthews correlation coefficients) for each model and the two types of classifier

Predictor set	Support vector machines	Extremely randomised trees
Brain volume	0.132 [0.115–0.148]	0.179 [0.164–0.194]
Number of lesions	0.168 [0.152–0.184]	0.229 [0.214–0.243]
Total lesion volume	0.229 [0.213–0.245]	0.239 [0.223–0.255]
Best low-dimensional	0.354 [0.339–0.369]	0.355 [0.341–0.369]
Regional atrophy	0.576 [0.563–0.590]	0.620 [0.607–0.633]
Regional disconnection	0.544 [0.530–0.559]	0.559 [0.545–0.573]
Best high-dimensional	0.666 [0.653–0.679]	0.675 [0.662–0.689]

All figures are given as the mean Matthews correlation coefficient [95% CI]

### Simulated, randomised, controlled trials

**Fig. 5:**
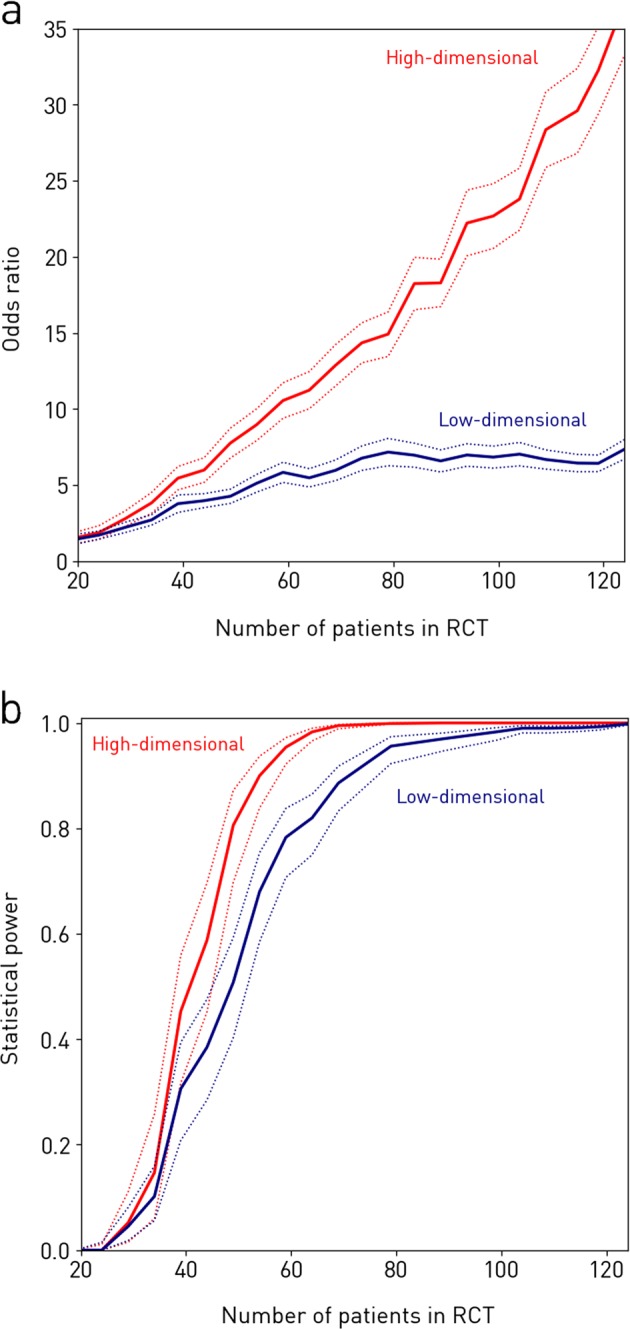
Simulated randomised controlled trials (RCT) of the intervention incorporating varying numbers of patients. Each RCT is simulated by randomly choosing a subset of *N* patients and evaluating the association between classifier output and imaging response with a Fisher’s exact test. The procedure is repeated 500 times, yielding a mean odds ratio (panel **a**, solid lines), and a mean achieved statistical power given a significance level of *α* = 0.01 (panel **b**, solid lines), along with 95% confidence interval (both panels, dotted lines) for each *N*. RCTs in red use the best performing high-dimensional model to detect the response, while RCTs in blue use the best performing low-dimensional model. **a** The odds ratio. It is seen that, if one performs an RCT with 124 patients enrolled, the odds ratio is 36.8 if one uses the high-dimensional method vs. only 7.35 if one uses a traditional low-dimensional approach. RCTs using the high-dimensional model not only produce larger odds ratios, but they also scale better with increasing patient numbers. **b** The achieved statistical power. We see that the statistical power achieved by the high- and low-dimensional models, respectively, are 0.806 [95% CI = 0.698–0.872] and 0.508 [95% CI = 0.403–0.593] with *N* = 50, at a significance level of *α* = 0.01. Thus in an RCT with 50 subjects enrolled, the risk of making a type II error is very significant with the traditional low-dimensional approaches, while a statistical power greater than 0.80 can be achieved at the same *N* with the high-dimensional method

In simulated randomised controlled trials (RCTs) of the intervention, the best high-dimensional model not only produced larger odds ratios, but the odds ratios also scaled better with increasing numbers of patients enrolled (Fig. 5a). Since the treatment effect size here was fixed, the improved odds ratios reflected better statistical efficiency. A linear fit to the data showed an increase in the odds ratio, for every additional patient enrolled, of 0.3 for the best high-dimensional model, compared with only 0.05 for the best, conventional, low-dimensional model. In particular, mean odds ratios with 59 patients enrolled were 5.83 (95% CIs: 5.17–6.49) and 10.6 (95% CIs: 9.38–11.7) for the conventional, low- and the proposed, high-dimensional approaches, respectively, diverging to 7.35 (95% CIs: 6.71–8.00) vs. 36.8 (95% CIs: 33.3–40.3) with 124 patients enrolled (Fig. 5a). As would be expected, enrolling more subjects in a given RCT increased the sensitivity with which an effect could be captured. However, Fig. 5a showed that this resulted in an early plateau in performance using conventional, low-dimensional methods, while the steep gradient in the odds ratio was strikingly persistent even at *N* = 124 with the proposed, high-dimensional methods. Therefore, while one could achieve higher sensitivity in an RCT by increasing the number of subjects enrolled, the benefits were much more limited with the conventional, low-dimensional approaches. The advantages of the proposed, high-dimensional methods were also apparent in the relation between the number of patients enrolled in the RCT and the power of the study (Fig. 5b). For example, here we saw that the statistical power achieved by the proposed, high- and the conventional, low-dimensional models, respectively, were 0.806 [95% CI = 0.698–0.872] compared with 0.508 [95% CI = 0.403–0.593] with *N* = 50, at a significance level of *α* = 0.01 (Fig. 5b). Thus, in an RCT with 50 subjects enrolled, the risk of making a type II error, in terms of hypothesis testing, was very significant with the conventional, low-dimensional approaches, while a statistical power greater than 0.80 could be achieved at the same *N* with the high-dimensional method. Although this difference became smaller when the number of subjects enrolled in the RCT was increased beyond 100, the individual-level prediction capability of the conventional, low-dimensional approaches lagged increasingly more behind the proposed, high-dimensional methods (Fig. 5a).

## Discussion

This study has revealed a substantial difference between the performance of conventional, low- and the proposed, high-dimensional models of imaging response to treatment in multiple sclerosis. We now turn to the interpretability, generalisability, and potential real-world applications of our results.

A natural concern when dealing with high-dimensional models is that they might ground their decision-making in imaging changes that are not physiologically meaningful. But neither changes in tissue loss nor degree of cortical disconnection can be easily misinterpreted, especially when they are shown to be reversals of familiar patterns of pathological change (Fig. 4). Pseudoatrophy, a phenomenon associated with immunomodulatory drugs such as natalizumab, occurs in the first 6 to 12 months of treatment, not over the greater than 5-year period here examined, and would be expected to produce the opposite pattern of change.^13–16^

Care must be taken in interpreting high-dimensional models of imaging response. Any imaging-based model will naturally be sensitive to changes in proportion to their visibility on MR, not their physiological impact. While the two go mostly hand-in-hand, especially when the signal of interest is rate of tissue loss or disconnection, it is important to establish an ultimate link to clinical outcomes. But this is feasible only after a sensitive and robust imaging response model is built, for a model trained directly on clinical outcomes would inevitably conflate the two sources of variability: that between treatment and imaging changes and that between imaging changes and measured clinical outcomes. Furthermore, a model of the imaging response to treatment is independently valuable in multiple sclerosis, indeed with any disseminated parenchymal pathology, owing to the many areas of life impact conventional clinical measured do not adequately capture.

The power of high-dimensional inference carries a risk: the danger of finding a solution that is not generalisable beyond the data on which the model has been trained. Contemporary machine learning counters this risk by using out-of-sample testing as the fundamental metric of performance, and algorithmic characteristics that penalise purely local solutions. Key aspects of our source data and modelling approach have further reduced this risk. First, our patient sample was not just large but also unselected, including everyone on our natalizumab register who has been treated at our centres over an interval of 15 years. Second, we deliberately kept our parameterisations of temporal change and of anatomy relatively crude. The trajectories used to estimate the former are dichotomised from purely linear models, and the anatomical features used to describe the latter are discretised into large brain regions. In the context of machine learning inference, the ratio of input features to number of cases was therefore kept conservative. All performance estimates were obtained with out-of-sample testing, and we included metrics robust to the class imbalance naturally observed within this clinical pathway. Demonstrating robust performance in the face of imbalanced data, without potentially unrealistic weighting or subsampling, strengthens the generalisability to real-world clinical contexts.

How well the approach will extend to other treatments in multiple sclerosis needs dedicated investigation, but since our input features capture the major aspects of imaging any treatment could conceivably modify—the patterns of lesional change and regional atrophy—similar performance can reasonably be expected. Indeed, where the treatment is less potent, or its effects dispersed over a wider interval, the integrative power of high-dimensional methods should amplify their benefit.

Detecting an imaging response to treatment is a foundational component of an array of possible applications with a focus either on individual patients or on population mechanistic or therapeutic aspects. Each presumes the prior availability of data of comparable range and scale to that achieved here, from which an adequately generalising model can be constructed, and against which new, unlabelled data can be tested. The insensitivity of our approach to scanner parameters—including the use of routine, clinical-grade acquisitions—enables the generation of such datasets by pooling heterogeneous data across disparate clinical units, without the need for costly and logistically difficult MR sequence optimisation. High-dimensional modelling can thus be readily brought into practice.

A question which can be addressed in future studies is whether the regional brain volume and disconnectome trajectories that were detected as the “most important” are truly the most important outcomes or are indirect measures of other changes or methodological issues such as the anisotropy of the clinical scans.

However, while high-quality, isotropic imaging is bound to produce better “fingerprints” of response and may alter the contribution of brain areas thereby more reliably quantified, imaging only practicable in specialist academic centres is here shown to be unnecessary (even if always desirable) and would likely maintain or amplify the advantage of our approach.

Where the pattern of detected change is convincingly an arrest or reversal of the pathological, it may be used as an independent measure of response to treatment in a specific patient, collaterally with clinical measures. Such a use is logically identical with counting the number of new lesions or other established radiologist-derived metrics, differing only in its sensitivity. As with any other radiological metric, corroboration by subsequent clinical evolution would be an important part of its development. Second, the pattern of detected change may be used to interpret individual clinical changes with greater precision. For example, where the pattern of cortical disconnection or atrophy is shown to impinge heavily on areas of the brain concerned with cognition, motor clinical measures are likely to be much less sensitive than cognitive ones, and ought to be commensurately weighted.

Determining the specific contribution of any one factor within a given population of patients naturally depends on our ability to model the many confounding factors that contribute to the outcome of interest. Where the variability introduced by confounders is treated simply as noise the sensitivity for detecting the effect of the factor we seek is commensurately reduced. In a randomised controlled trial of an intervention, a better model of the imaging outcome thus enhances our ability to detect a true imaging response to treatment, and should be used where this is an independent outcome of interest. Such a model would also enhance our ability to identify incidental factors that interact with the treatment, enabling us better to identify biological features that modulate treatment responsiveness. Where the outcome of interest is purely clinical, capturing its high-dimensional imaging correlates allows us to weight the predicted clinical eloquence of the treatment effect, enhancing its detection. The imaging response is here used to deconfound the clinical from the interaction between disease patterns and the underlying functional anatomy. Naturally, these applications will require more than the imaging response models on which they are all founded, but our objective here is to outline the horizon of possibilities our general approach discloses.

The clinical implication of applying high-dimensional modelling to predict the imaging response to a treatment to patients with multiple sclerosis is that individual patients may be counselled about their probability of responding to a specific therapy prior to treatment initiation, or soon after, thereby increasing the possibility of initiating the most effective treatment for their individual disease profile. Pharmacological companies may use the individual prediction of treatment response provided by this technology to select patients who are more likely to respond to a specific therapy, with consequent reduction in the sample size and cost of the trial. Therefore, the future impact of this technology is to improve patient management and treatment, and facilitate the testing of drug efficacy and monitoring of treatment response.

In conclusion, this study demonstrates the value of modelling routine imaging data with high-dimensional techniques that closely reflect the complexity of the brain, not only as a tool for illuminating diseases mechanisms, but as a practical, real-world means of determining the power of therapeutic interventions to change them, both for individual patient care and interventional studies.

## Methods

### Patients and MRI

We identified relapsing–remitting multiple sclerosis patients treated with natalizumab at the National Hospital for Neurology and Neurosurgery, University College London Hospitals NHS Foundation Trust, London, UK, according to local, standard-of-care protocols, for whom at least one full-brain scan pre- or post-initiation of natalizumab was available. This yielded imaging data from 124 patients (563 scans from 2001 to 2016), acquired using routine, clinical MRI protocols with six different 1.5 T or 3 T scanners (Siemens, Philips, and GE), all of which were irrevocably anonymised. Clinical characteristics of the patients corresponded to the standard clinical indications for initiating natalizumab in the UK, where natalizumab is licensed as a second-line treatment, and were typical of such cohorts (Table 6, Supplementary Table 1).

**Table 6:** Patient demographics and clinical characteristics

Number of patients (*N*)	124
Gender	44 M, 80 F
Age, years^a,b^	38 (20–70)
Weight, kg^b^	70.3 (45–111)
Disease duration, years^a,c^	6.3 (2.2)
EDSS^a,c^	4.8 (1.7)
Length of follow-up, years^c^	4.2 (2.6)
Previous therapy^d^	63 (50.8%)—beta interferons
	31 (25.0%)—beta interferons and copaxone
	14 (11.3%)—copaxone
	16 (12.9%)—other medications/medicine combinations

^a^At the start of treatment

^b^Mean (range)

^c^Mean (standard deviation)

^d^Number (%) of patients

Our analysis used T1-weighted and fluid-attenuated inversion recovery (FLAIR) scans, having an average voxel resolution of 1 × 1 × 6 mm^3^, which is typical of routine, standard-of-care, brain MR imaging in the UK. Acquisition and other instrumental parameters varied both across and within patients.

The study had ethical and institutional approval at University College London Hospitals NHS Foundation Trust for consentless analysis of fully-anonymised, routinely collected data and was performed in accordance with the relevant guidelines and regulations.

### Image processing and feature extraction

**Fig. 6:**
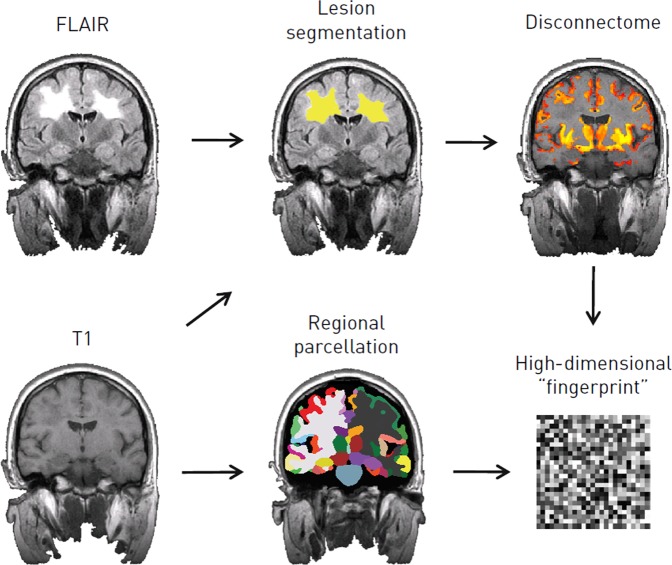
Extracting a high-dimensional imaging “fingerprint”. The T1-weighted MRI volume is used to obtain the regional parcellation of the brain, and alongside the FLAIR to detect white matter lesions. The brain “disconnectome”, which is subsequently obtained using the detected lesion mask, is a probabilistic map of regional disconnection. The degree of disconnection is colour coded, so that grey matter regions that appear more yellow in the disconnectome map are predicted to have higher degrees of disconnection, due to the presence of the multiple sclerosis lesions in the white matter tracts that are connected to them, than regions which appear in red. The output from all these steps, following slope and trajectory calculation, is a high-dimensional imaging “fingerprint” comprising the trajectories of the normalised regional brain volumes and the degrees of disconnection for each of the regions in the brain parcellation

To construct models of imaging response, we extracted a rich, multivariate set of parameters from each image—an imaging “fingerprint”—and monitored their evolution over the available time series. To minimise bias and maximise practicability, all processing was fully automated.^17^ We implemented well-established solutions to each of the three necessary steps: anatomical registration and regional brain parcellation, lesion segmentation and brain disconnection estimation (“disconnectome”) (Fig. 6). In brief, the Geodesic Information Flows (GIF) method^18^ was applied to each T1-weighted image to generate a set of 144 regions-of-interest, both natively and in standard Montreal Neurological Institute 152 (MNI152) stereotactic space. The GIF method has previously been successfully used for the parcellation of anisotropic brain MR images.^7,19,20^

In parallel, focal white matter lesions were segmented from the FLAIR images in conjunction with the T1 data using a dual-modality approach, combining patch match^21^ and expectation maximisation algorithms,^22^ also both natively and in MNI152 space. The resultant lesion masks were checked and validated by a neurologist (RC) and a neuroradiologist (FB): all were found to be satisfactory. Finally, we computed whole-brain, voxel-wise, probabilistic maps of disconnection in MNI152 space^23–25^ using the Brain Connectivity and Behaviour toolkit.^26^ This toolkit estimates the probability of disconnection of each brain region by combining the localisation of the white matter damage with a probabilistic tractographic atlas. Each white matter lesion is thus projected to the brain regions connected by the tracts it disrupts (see Fig. 6).

The outputs from these steps were total lesion volume, number of lesions, total brain volume, and a high-dimensional imaging “fingerprint” of 288 variables, incorporating regional brain volumes within the GIF parcellation, and their estimated degrees of disconnection (Table 7).

**Table 7:** Processing steps and the parameters derived

Processing step	Input	Output
Regional parcellation	T1	144 regional brain volumes, total brain volume
Lesion segmentation	T1 & FLAIR	Total lesion volume, number of lesions
Disconnectome estimation	Lesion segmentation	144 regional brain disconnection estimates

### Confounder removal

To facilitate the extraction of the signal of interest—the specific effect of the intervention—it is desirable to minimise variability arising from other factors with a bearing on the appearance of the brain. The covariates of age, gender, scanner manufacturer, magnetic field strength, disease duration, Expanded Disability Status Scale (EDSS) score, and voxel size (T1 and FLAIR) were therefore regressed out of all the imaging-derived parameters using BayesReg.^27^ Such cofounder removal is kin with the covariance adjustment widely used in trials and observational studies.^28^ Total lesion volume, total brain volume and regional brain volumes were normalised by the total intracranial volume. To confirm that the confounders were successfully removed,^29,30^ we separately fitted multivariate models attempting to predict them and confirmed that they could not. All subsequent analyses used regional and global parameters deconfounded in this way.

### Longitudinal feature extraction

**Fig. 7:**
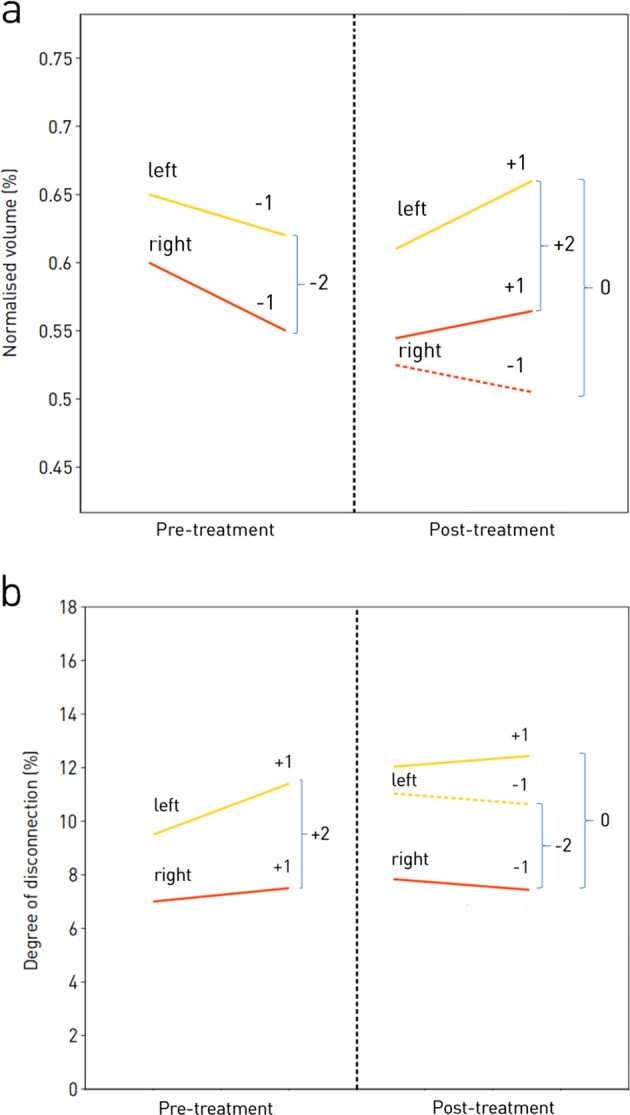
Illustration of the slope of an imaging metric between two pre-treatment scans, and the different potential slopes between two subsequent post-treatment scans, showing how these are scored. **a** In the pre-treatment period the imaging metric, e.g. the normalised volume of the thalamus is decreasing in both the left and the right hemispheres, thus each has a trajectory of −1, leading to a net trajectory of −2. In the post-treatment period, the normalised volume of the left thalamus is increasing, while we illustrate two differential scenarios: one in which the normalised volume of the right thalamus is continuing to decrease, and one which the normalised volume is increasing, leading to net trajectories for the normalised volume of the thalamus of 0, and +2, respectively. **b** In the pre-treatment period the imaging metric, e.g. the degree of disconnection of the thalamus is increasing in both the left and the right hemispheres, thus each has a trajectory of 1, leading to a net trajectory of +2. In the post-treatment period, the degree of disconnection of the right thalamus is getting smaller, while we illustrate two differential scenarios: one in which the degree of disconnection of the left thalamus is continuing to increase, and one which the degree of disconnection is getting smaller, leading to net trajectories for the degree of disconnection of the thalamus of 0, and −2, respectively

Our objective is to detect a difference—before and after the start of treatment—of the *rate of change* of the imaging-derived parameters. The rate of change is simply quantified by the *slope* of a line fitted to two or more points dispersed over time. We therefore performed—independently for each variable—linear regression on the sets of available scans before and after the start of treatment, taking the slope as a measure of the rate of change of the relevant parameter over time (Fig. 7). For example, the normalised volume of the thalamus, indexed on each of the pre-treatment scans, was linearly regressed against time to yield a pre-treatment slope, and the same was done on the post-treatment scans to yield a post-treatment slope. The process was replicated for each area and each patient, and for both volumes and disconnections, yielding two sets of slopes for each patient—before and after treatment—for each of the 288 variables and their derived low-dimensional aggregates. For some patients only a pre-treatment (*N* = 16) or post-treatment (*N* = 60) slope was available for reasons of data non-availability.

The accuracy of slope estimates will inevitably vary with the number and timing of individual scans. To minimise any resultant confounding effects, we converted each slope to a trajectory by taking its sign, yielding +1 for a positive slope, and −1 for a negative slope (Fig. 7). Where a slope is exactly 0, e.g. for ordinal or bounded variables, a third class denoting no change (slope = 0) was introduced. Note that the cost of minimising potential bias here is loss of some information on the relatively reliability of each slope estimate, reducing our ability to reject noise arising from variability over short intervals. A potential reduction in sensitivity is, however, preferable to retaining information that might be a source of bias. Equally, though greater sensitivity could be achieved by explicitly modelling global, disease non-specific effects of an intervention such as pseudoatrophy, the risk of introducing bias motivates us to desist.

Since only relatively weak, population-level lateralisation of the impact of multiple sclerosis on the brain has been observed, and there are no grounds for expecting lateralised treatment effects, we collapsed trajectories across homologous regions of each hemisphere, taking the sum of the trajectories for each pair. This increases the compactness of our models without likely substantial loss of information.

### Models of imaging response

To quantify the effect of dimensionality on our ability to detect an imaging response to treatment, we evaluated the performance of conventional, low- and the proposed, high-dimensional models in detecting whether a set of trajectories belonged to the pre- or post-treatment period. The outcome was, therefore, our ability to differentiate between measurements made in the pre- vs. post-treatment period. We used two different classifiers: support vector machines (SVM)^31^ and extremely randomised trees (ERT)^32^ implemented in scikit-learn.^33^ They were chosen as representative of two different, widely used architectures that exhibit different degrees of flexibility, the latter greater than the former, allowing us to explore the effects of maximal model capacity independently of the input dimensionality.

Cross-validation was performed using bootstrapping (500 iterations), fully holding-out each test set, and randomly allocating 80% of the cases for training and 20% for testing at each iteration (Supplementary code snippet 1). The randomisation and bootstrapping were carried out at the subject level and were constrained to prevent the selection of the same patient’s data for both training and testing at the same time, in the case of patients who had both pre- and post-natalizumab trajectories available (*N* = 27). The parameters of the two classifiers were tuned manually (with the same bootstrapped, out-of-fold, cross-validated setup described above and shown in Supplementary code snippet 1), the final settings are given in Table 8.

**Table 8:** Final parameters of the two types of machine learning classifiers used

Classifier	Final parameters and settings
SVM	Gaussian radial basis function kernel, penalty term C = 10 (with balanced class weighting), kernel coefficient *γ* = number of features^−1^
ERT	Gini impurity as the tree-splitting metric, number of trees = 100, number of features to consider when looking for the best split Mf = number of features^1/2^, balanced class weighting

*SVM* support vector machines, *ERT* extremely randomised trees

### Feature and model selection

We used the greedy, forward-stepwise feature selection algorithm shown in Supplementary code snippet 2 which utilises the mean bootstrapped, out-of-fold, cross-validation AUC as the performance measure. This approach is preferable over the use of feature importances that can be obtained from classifiers such as randomised trees. Unlike the latter which measure feature importances based on the in-fold data they see during training, the importance of each feature in our method is measured using bootstrapped, out-of-fold, cross-validation, thus giving unbiased performance estimates.

The selection of low- and high-dimensional features, and in the case of the latter, the selection of regional volume, and regional disconnectome features were carried out separately. Our final predictors were then the trajectories of the number of lesions, total lesion volume, and total brain volume (for the conventional, low-dimensional case), and a high-dimensional “imaging fingerprint”, which incorporated the trajectories of 23 lesion- and 20 tissue-derived features extracted from MR images acquired at pre- or post-treatment patient visits.

Once the selection of regional volume and the disconnectome features was completed, the best high-dimensional model was then constructed as one which provided a weighted average of the predictions made by the best high-dimensional model based solely on regional brain volume trajectories, and the best high-dimensional model based solely on regional brain disconnectome trajectories. This approach was chosen over one which incorporates both dimensions simultaneously as it allows us to test the performance of models that incorporate only volumetric and disconnectome metrics, while providing a clearer understanding of which volumetric and disconnectome measures contribute more to imaging response detection. Feature selection was carried out once and the same features were subsequently used throughout.

### Performance evaluation

Receiver operating characteristic (ROC) curves were used to quantify the detection performance of each model, qualified statistically by the area under the ROC curve (AUC), accuracy, sensitivity and specificity. Two-sided, two-sample Kolmogorov–Smirnov tests were used for statistical validation and significance values less than 0.05 were considered statistically significant. Performance values were aggregated with respect to bootstraps in terms of the mean and the standard error.

We also investigated the performance of conventional, low-dimensional and the proposed, high-dimensional models to detect the imaging response to treatment in simulated, RCTs of the intervention enrolling varying numbers of patients (ranging from 19 to 124 in number). Each RCT was simulated by randomly choosing a subset of *N* patients and evaluating the association between classifier output and imaging response with Fisher’s exact test. The procedure was repeated 500 times, yielding for each *N* a mean odds ratio and an achieved power given a significance threshold of *α* = 0.01, both with 95% confidence intervals. Statistical power calculations were performed with the G*Power^34^ software.

### Reporting Summary

Further information on research design is available in the Nature Research Reporting Summary linked to this article.

## Supplementary information

Supplementary Material

Reporting Summary

## Data Availability

Due to ethical concerns, supporting data cannot be made openly available. Further information about the data that support the findings of this study is available from the corresponding author upon reasonable request.

## Supplementary information

**Supplementary information** accompanies the paper on the *npj Digital Medicine* website (10.1038/s41746-019-0127-8).

## Code Availability

The software used in this study were as follows: Python 2.7 (https://www.python.org/), scikit-learn 0.19.2 (https://scikit-learn.org), Numpy 1.15.4 (http://www.numpy.org/), Scipy 1.1.0 (https://www.scipy.org/), Brain Connectivity and Behaviour toolkit 2018 (http://toolkit.bcblab.com/), GIF v3 (http://niftyweb.cs.ucl.ac.uk/program.php?p=GIF), BayesReg 1.8.0.1 (https://www.mathworks.com/matlabcentral/fileexchange/60823-bayesian-penalized-regression-with-continuous-shrinkage-prio), G*Power 3 (http://www.gpower.hhu.de/), MATLAB 2018 (https://www.mathworks.com), NiftyReg (https://github.com/KCL-BMEIS/niftyreg/wiki). All software are open-source/publicly available, except for GIF v3, and MATLAB, which are commercial packages.

## References

[1] Burgess PW, Alderman N, Evans J, Emslie H, Wilson BA (1998). The ecological validity of tests of executive function. Int. J. Neuropsychol. Soc..

[2] Eshaghi A (2018). Deep gray matter volume loss drives disability worsening in multiple sclerosis. Ann. Neurol..

[3] Wattjes MP (2015). Evidence-based guidelines: MAGNIMS consensus guidelines on the use of MRI in multiple sclerosis–establishing disease prognosis and monitoring patients. Nat. Rev. Neurol..

[4] Rovira À (2015). Evidence-based guidelines: MAGNIMS consensus guidelines on the use of MRI in multiple sclerosis—clinical implementation in the diagnostic process. Nat. Rev. Neurol..

[5] Enzinger C (2015). Nonconventional MRI and microstructural cerebral changes in multiple sclerosis. Nat. Rev. Neurol..

[6] Steenwijk MD (2015). Cortical atrophy patterns in multiple sclerosis are non-random and clinically relevant. Brain.

[7] Eshaghi A (2018). Progression of regional grey matter atrophy in multiple sclerosis. Brain.

[8] Xu T, Rolf Jäger H, Husain M, Rees G, Nachev P (2018). High-dimensional therapeutic inference in the focally damaged human brain. Brain.

[9] Yoo, Y. et al. Deep learning of brain lesion patterns and user-defined clinical and MRI features for predicting conversion to multiple sclerosis from clinically isolated syndrome. *Comput. Methods Biomech. Biomed. Eng. Imaging Vis.* **0**, 1–10 (2017).

[10] Yoo Youngjin, Tang Lisa W., Brosch Tom, Li David K. B., Metz Luanne, Traboulsee Anthony, Tam Roger (2016). Deep Learning of Brain Lesion Patterns for Predicting Future Disease Activity in Patients with Early Symptoms of Multiple Sclerosis. Deep Learning and Data Labeling for Medical Applications.

[11] Doyle, A., Precup, D., Arnold, D. L. & Arbel, T. Predicting future disease activity and treatment responders for multiple sclerosis patients using a bag-of-lesions brain representation. In *Medical Image Computing and Computer-Assisted Intervention* (eds Descoteaux, M. et al.) 186–194 (Springer International Publishing, Cham, Switzerland, 2017).

[12] Brosch, T., Yoo, Y., Li, D. K. B., Traboulsee, A. & Tam, R. Modeling the variability in brain morphology and lesion distribution in multiple sclerosis by deep learning. in *Medical Image Computing and Computer-Assisted Intervention* (eds Golland, P., Hata, N., Barillot, C., Hornegger, J. & Howe, R.) 462–469 (Springer International Publishing, Cham, Switzerland, 2014). 10.1007/978-3-319-10470-6_58 25485412

[13] Sastre-Garriga J (2015). Brain atrophy in natalizumab-treated patients: a 3-year follow-up. Mult. Scler..

[14] Filippi M (2012). Association between pathological and MRI findings in multiple sclerosis. Lancet Neurol..

[15] Favaretto A, Lazzarotto A, Margoni M, Poggiali D, Gallo P (2018). Effects of disease modifying therapies on brain and grey matter atrophy in relapsing remitting multiple sclerosis. Mult. Scler. Demyelinating Disord..

[16] Vidal-Jordana A (2013). Early brain pseudoatrophy while on natalizumab therapy is due to white matter volume changes. Mult. Scler..

[17] Kanber, B. et al. An integrated imaging informatics software platform to improve the analysis of clinical trials and research data in MS. In *Proc. 32nd Congress of the European Committee for Treatment and Research in Multiple Sclerosis* Vol. 22, 229–230 (London, England, 2016).

[18] Cardoso MJ (2015). Geodesic information flows: spatially-variant graphs and their application to segmentation and fusion. IEEE Trans. Med. Imaging.

[19] Lesjak Ž (2018). A nNovel public MR image dataset of multiple sclerosis Patients with lesion segmentations based on multi-rater consensus. Neuroinformatics.

[20] Kieselmann JP (2018). Geometric and dosimetric evaluations of atlas-based segmentation methods of MR images in the head and neck region. Phys. Med. Biol..

[21] Prados, F. et al. Multi-contrast patchmatch algorithm for multiple sclerosis lesion detection. In: *ISBI 2015 – Longitudinal MS Lesion Segmentation Challenge*. pp. 1–2 (2015). https://www.ncbi.nlm.nih.gov/pmc/articles/PMC5344762/.

[22] Sudre CH (2015). Bayesian model selection for pathological neuroimaging data applied to white matter lesion segmentation. IEEE Trans. Med. Imaging.

[23] Thiebaut de Schotten M (2011). A lateralized brain network for visuospatial attention. Nat. Neurosci..

[24] Thiebaut de Schotten M (2015). From Phineas Gage and Monsieur Leborgne to H.M.: revisiting disconnection syndromes. Cereb. Cortex.

[25] Rojkova K (2016). Atlasing the frontal lobe connections and their variability due to age and education: a spherical deconvolution tractography study. Brain Struct. Funct..

[26] Foulon C (2018). Advanced lesion symptom mapping analyses and implementation as BCBtoolkit. GigaScience.

[27] Makalic, E. & Schmidt, D. F. High-dimensional Bayesian regularised regression with the BayesReg Package. Preprint at arXiv (2016). https://arxiv.org/abs/1611.06649.

[28] Rosenbaum PR (2002). Covariance adjustment in randomized experiments and observational studies. Stat. Sci..

[29] Snoek, L., Miletić, S. & Scholte, H. S. How to control for confounds in decoding analyses of neuroimaging data. *bioRxiv* 290684 (2018). 10.1101/290684. 10.1016/j.neuroimage.2018.09.074 30268846

[30] Linn KA, Gaonkar B, Doshi J, Davatzikos C, Shinohara RT (2016). Addressing confounding in predictive models with an application to neuroimaging. Int. J. Biostat..

[31] Chang C-C, Lin C-J (2011). LIBSVM: A Library for Support Vector Machines. ACM Trans. Intell. Syst. Technol..

[32] Geurts P, Ernst D, Wehenkel L (2006). Extremely randomized trees. Mach. Learn..

[33] Pedregosa F (2011). Scikit-learn: Machine Learning in Python. J. Mach. Learn. Res..

[34] Faul F, Erdfelder E, Lang A-G, Buchner A (2007). G*Power 3: a flexible statistical power analysis program for the social, behavioral, and biomedical sciences. Behav. Res. Methods.

