# Author Correction: High-dimensional detection of imaging response to treatment in multiple sclerosis

**DOI:** 10.1038/s41746-019-0144-7

**Published:** 2019-07-16

**Authors:** Baris Kanber, Parashkev Nachev, Frederik Barkhof, Alberto Calvi, Jorge Cardoso, Rosa Cortese, Ferran Prados, Carole H. Sudre, Carmen Tur, Sebastien Ourselin, Olga Ciccarelli

**Affiliations:** 1 Centre for Medical Image Computing, Department of Medical Physics and Biomedical Engineering, University College London, London, UK; 2 Multiple Sclerosis Research Centre, NMR Research Unit, Department of Neuroinflammation, Queen Square Institute of Neurology, University College London, London, UK; 3 National Institute for Health Research (NIHR), University College London Hospitals Biomedical Research Centre (BRC), London, UK; 4 High Dimensional Neurology Group, Queen Square Institute of Neurology, University College London, London, UK; 5 Department of Radiology & Nuclear Medicine, VU University Medical Center, Amsterdam, The Netherlands; 6 School of Biomedical Engineering & Imaging Sciences, King’s College London, London, UK

**Keywords:** Multiple sclerosis, Translational research

Correction to: *npj Digital Medicine* 10.1038/s41746-019-0127-8, published online 10 June 2019

The original version of the published Article listed the incorrect affiliations for Jorge Cardoso, Carole H. Sudre, and Sebastien Ourselin. These authors’ affiliations are now correctly noted as School of Biomedical Engineering & Imaging Sciences, King’s College London, London, UK. Additionally, a statement from the Acknowledgments section was omitted. The Acknowledgments have been updated to include the following: “We are grateful to the MS Society for its support of the Multiple Sclerosis Research Centre at the Queen Square Institute of Neurology where this work was completed.” The HTML and PDF versions of the Article have been corrected.

