# Supplementary material for: High-dimensional detection of imaging response to treatment in multiple sclerosis
Source: NPJ Digit Med. 2019 Jun 10;2:49. doi: 10.1038/s41746-019-0127-8 (PMC6556513; doi:10.1038/s41746-019-0127-8)
Supplement: Supplementary file 1 — Supplementary Material [file 41746_2019_127_MOESM1_ESM.docx]

**Supplementary material**

**Class imbalance**

We confirmed the stability of our results with respect to class imbalance by subsampling the available data to obtain a balanced subset, comparing the resulting predictive capabilities of the low- and high-dimensional ERT models with those obtained in the main experiment.

**Subsampled classes**

**Class 0:** 40 samples

**Class 1:** 41 samples

**AUC [95% CI] for the best low-dimensional ERT model:** 0.666 [0.658-0.675] compared with 0.686 [0.679-0.693] obtained in the main experiment.

**AUC [95% CI] for the best high-dimensional ERT model:** 0.869 [0.863-0.876] compared with 0.890 [0.885-0.895] in the main experiment.

Since subsampling inevitably reduces the amount of training data, a reduction in performance was observed. But this was small―AUC decrement of 0.021 for the best high-dimensional ERT model, and 0.020 for the best low-dimensional ERT model― and crucially left the performance gap between the high- and low-dimensional models essentially unchanged (AUC difference: 0.203 after subsampling, compared with 0.204 in the main experiment). Therefore, as suggested by performance metrics insensitive to it, class imbalance cannot explain the substantial advantage of high-dimensional modelling observed here.

**Supplementary Table 1 – Clinical details and timing of scans in the pre- and post-treatment periods.** The mean time interval between scans ranged between 0.1 and 5.8 years for the pre-treatment period, and between 0.4 and 4.1 years for the post-treatment period. Values for age, Expanded Disability Status Scale (EDSS) score, and disease duration (DD) are given as at the start of treatment with natalizumab.

| **Subject** | **Age (years)** | **Previous DMT^σ^** | **EDSS** | **DD (years)** | **Number of scans** | | **Average time between scans (years)** | |
| --- | --- | --- | --- | --- | --- | --- | --- | --- |
|  |  |  |  |  | **Pre** | **Post** | **Pre** | **Post** |
| 1 | 48 | CPx | 4.5 | 5.2 | 0 | 3 |  | 1.8 |
| 2 | 28 | INTB 1a | 3 | 7.0 | 1 | 4 |  | 1.1 |
| 3 | 28 | INTB 1a | 2 | 4.0 | 2 | 6 | 0.8 | 1 |
| 4 | 27 | CPx | 2 | 7.3 | 3 | 0 | 2.9 |  |
| 5 | 26 | INTB 1b | 3 | 7.5 | 1 | 2 |  | 1 |
| 6 | 38 | CPx | 5 | 7.5 | 1 | 1 |  |  |
| 7 | 28 | INTB 1b | 2 | 6.8 | 1 | 4 |  | 0.7 |
| 8 | 47 | INTB 1a | 3.5 | 8.2 | 0 | 1 |  |  |
| 9 | 44 | INTB 1a, CPx | 3 | 3.8 | 0 | 5 |  | 1.5 |
| 10 | 34 | INTB 1a | 2 | 6.4 | 0 | 3 |  | 0.5 |
| 11 | 27 | INTB 1a | 6.5 | 2.7 | 1 | 4 |  | 1.6 |
| 12 | 28 | INTB 1a | 6 | 4.4 | 2 | 4 | 1.2 | 1.7 |
| 13 | 38 | INTB 1a | 6 | 2.9 | 1 | 7 |  | 0.8 |
| 14 | 45 | CPx | 6.5 | 4.0 | 1 | 5 |  | 1.1 |
| 15 | 34 | INTB 1a | 6 | 6.1 | 2 | 4 | 0.8 | 1 |
| 16 | 45 |  | 4 | 7.5 | 0 | 3 |  | 1.2 |
| 17 | 49 |  | 3 | 9.7 | 4 | 1 | 1.8 |  |
| 18 | 41 | INTB 1a | 3.5 | 5.4 | 2 | 9 | 1.3 | 0.6 |
| 19 | 24 | INTB 1a | 4 | 6.5 | 2 | 2 | 1.1 | 1.5 |
| 20 | 42 | INTB 1a | 3 | 5.6 | 1 | 5 |  | 0.8 |
| 21 | 23 | INTB 1a | 5 | 5.6 | 0 | 3 |  | 1.5 |
| 22 | 21 | INTB 1a | 1 | 7.7 | 1 | 3 |  | 1.1 |
| 23 | 38 | INTB 1a | 3 | 4.4 | 0 | 6 |  | 0.7 |
| 24 | 37 | INTB 1a | 4 | 7.4 | 2 | 3 | 0.2 | 0.4 |
| 25 | 47 | INTB 1a | 7 | 3.5 | 1 | 7 |  | 0.8 |
| 26 | 46 | INTB 1a | 3 | 7.6 | 1 | 1 |  |  |
| 27 | 40 | TERi | 4 | 5.6 | 0 | 2 |  | 2.9 |
| 28 | 30 |  | 6 | 7.5 | 1 | 1 |  |  |
| 29 | 27 | CPx, INTB 1a | 2.5 | 4.8 | 1 | 5 |  | 1 |
| 30 | 54 | INTB 1a | 6 | 3.3 | 1 | 4 |  | 2 |
| 31 | 39 | INTB 1a, CPx | 6 | 5.6 | 1 | 6 |  | 0.8 |
| 32 | 57 | INTB 1a | 3 | 6.6 | 1 | 3 |  | 1 |
| 33 | 44 | INTB 1a, CPx | 4 | 7.7 | 1 | 3 |  | 1 |
| 34 | 20 | CPx, INTB 1a | 2 | 5.8 | 1 | 5 |  | 1 |
| 35 | 43 | CPx | 2 | 9.7 | 3 | 0 | 1.2 |  |
| 36 | 24 | CPx | 4 | 2.9 | 1 | 6 |  | 1.1 |
| 37 | 39 | INTB 1a | 6 | 9.7 | 4 | 0 | 0.7 |  |
| 38 | 42 | CPx | 6 | 4.0 | 0 | 4 |  | 1.1 |
| 39 | 38 |  | 6 | 4.1 | 1 | 8 |  | 0.7 |
| 40 | 32 | INTB 1a | 3 | 2.8 | 0 | 8 |  | 0.9 |
| 41 | 67 | INTB 1a | 6 | 7.7 | 2 | 1 | 0.8 |  |
| 42 | 35 | INTB 1a | 3.5 | 6.8 | 0 | 4 |  | 0.7 |
| 43 | 44 | INTB 1a, CPx | 6.5 | 3.9 | 1 | 7 |  | 0.9 |
| 44 | 49 | INTB 1a | 6.5 | 5.4 | 0 | 6 |  | 0.9 |
| 45 | 33 | INTB 1a | 7.5 | 6.1 | 4 | 5 | 0.9 | 0.9 |
| 46 | 25 | INTB 1a, CPx | 6 | 2.7 | 3 | 4 | 0.5 | 1.9 |
| 47 | 38 | INTB 1a, CPx | 2 | 8.0 | 2 | 0 | 1.9 |  |
| 48 | 42 | INTB 1a | 6.5 | 2.3 | 0 | 6 |  | 1.1 |
| 49 | 36 | INTB 1a | 6.5 | 4.7 | 1 | 3 |  | 0.9 |
| 50 | 47 | INTB 1a, CPx | 6 | 8.5 | 1 | 2 |  | 1 |
| 51 | 46 | INTB 1a, CPx | 6 | 3.8 | 1 | 4 |  | 1.4 |
| 52 | 48 | INTB 1a | 6 | 3.7 | 2 | 5 | 0.5 | 0.9 |
| 53 | 32 | INTB 1a | 6.5 | 5.6 | 2 | 5 | 0.4 | 1 |
| 54 | 24 | INTB 1a | 2 | 6.2 | 0 | 1 |  |  |
| 55 | 48 | INTB 1a | 6.5 | 4.3 | 1 | 8 |  | 0.8 |
| 56 | 20 | INTB 1a, CPx | 5 | 8.6 | 0 | 1 |  |  |
| 57 | 25 | INTB 1a, CPx | 4 | 6.8 | 2 | 2 | 2.5 | 2 |
| 58 | 46 | INTB 1a | 3 | 5.7 | 2 | 5 | 1.3 | 0.4 |
| 59 | 28 | INTB 1a | 6 | 4.2 | 1 | 11 |  | 0.6 |
| 60 | 33 | INTB 1a | 2.5 | 6.8 | 3 | 3 | 2.9 | 0.8 |
| 61 | 29 | CPx | 5.5 | 7.0 | 2 | 3 | 1.8 | 1 |
| 62 | 26 |  | 4 | 6.9 | 4 | 1 | 0.2 |  |
| 63 | 48 | INTB 1a | 4 | 5.2 | 1 | 4 |  | 1.1 |
| 64 | 68 | INTB 1a, CPx | 6 | 6.7 | 4 | 4 | 0.6 | 1 |
| 65 | 20 | INTB 1a | 6 | 8.5 | 1 | 1 |  |  |
| 66 | 40 | INTB 1a | 6 | 6.4 | 1 | 2 |  | 1.9 |
| 67 | 36 | INTB 1a, CPx | 2 | 5.5 | 0 | 5 |  | 0.7 |
| 68 | 64 | INTB 1a, CPx | 6 | 3.1 | 1 | 7 |  | 1 |
| 69 | 32 | INTB 1a, CPx | 3.5 | 2.5 | 0 | 5 |  | 1 |
| 70 | 38 | CPx | 5.5 | 6.4 | 0 | 5 |  | 1 |
| 71 | 52 | INTB 1a | 7 | 9.8 | 1 | 1 |  |  |
| 72 | 32 | CPx | 6 | 7.1 | 1 | 2 |  | 0.5 |
| 73 | 32 | INTB 1a | 5.5 | 4.2 | 1 | 2 |  | 0.5 |
| 74 | 26 | INTB 1a | 1.5 | 4.2 | 3 | 7 | 0.8 | 0.9 |
| 75 | 30 | INTB 1a | 7 | 4.5 | 1 | 3 |  | 0.7 |
| 76 | 33 | INTB 1a | 6 | 5.0 | 0 | 6 |  | 0.8 |
| 77 | 47 | INTB 1a | 3 | 4.2 | 2 | 3 | 1.4 | 0.9 |
| 78 | 27 | AZA | 6 | 8.7 | 2 | 1 | 1.7 |  |
| 79 | 70 | INTB 1a | 3 | 8.4 | 2 | 2 | 2 | 0.8 |
| 80 | 47 | INTB 1a | 6 | 5.0 | 1 | 6 |  | 0.7 |
| 81 | 41 |  | 8 | 8.8 | 2 | 1 | 3.4 |  |
| 82 | 30 | INTB 1a, CPx | 6 | 3.1 | 3 | 5 | 0.7 | 1.5 |
| 83 | 33 | INTB 1a, CPx | 4.5 | 3.5 | 1 | 4 |  | 0.8 |
| 84 | 28 |  | 6 | 4.9 | 8 | 3 | 0.1 | 2.3 |
| 85 | 37 | CPx | 4 | 9.6 | 1 | 0 |  |  |
| 86 | 65 | INTB 1a, CPx | 5 | 3.5 | 2 | 8 | 0.4 | 0.8 |
| 87 | 35 | INTB 1a, CPx, FIN | 3 | 9.1 | 2 | 0 | 4 |  |
| 88 | 24 | INTB 1a, CPx | 7 | 3.1 | 3 | 3 | 0.1 | 2 |
| 89 | 26 | INTB 1a, CPx | 2 | 2.9 | 2 | 2 | 0.6 | 0.9 |
| 90 | 54 | CPx | 7 | 9.1 | 3 | 1 | 2.4 |  |
| 91 | 39 | INTB 1a | 6.5 | 4.7 | 1 | 8 |  | 0.7 |
| 92 | 46 | CPx | 6 | 6.9 | 1 | 2 |  | 1.5 |
| 93 | 37 | INTB 1a, CPx | 5.5 | 8.1 | 0 | 2 |  | 0.9 |
| 94 | 24 | INTB 1a, CPx | 2 | 9.4 | 2 | 0 | 1.7 |  |
| 95 | 44 | INTB 1a, CPx | 6.5 | 7.1 | 0 | 6 |  | 0.4 |
| 96 | 47 | INTB 1a, CPx | 5 | 7.0 | 3 | 2 | 1.7 | 0.9 |
| 97 | 58 | INTB 1a | 6 | 8.1 | 0 | 2 |  | 0.9 |
| 98 | 43 | INTB 1a | 7 | 7.1 | 0 | 1 |  |  |
| 99 | 23 | INTB 1a | 6.5 | 3.9 | 1 | 7 |  | 0.9 |
| 100 | 25 | INTB 1a | 6 | 2.5 | 1 | 5 |  | 0.9 |
| 101 | 40 | INTB 1a, CPx | 5 | 6.0 | 1 | 2 |  | 2.5 |
| 102 | 46 | INTB 1a, CPx | 6 | 6.5 | 0 | 1 |  |  |
| 103 | 22 | INTB 1a | 4.5 | 5.4 | 3 | 2 | 0.8 | 3.1 |
| 104 | 41 | INTB 1a | 7 | 3.7 | 2 | 4 | 0.6 | 1.5 |
| 105 | 42 | INTB 1a, CPx | 6 | 8.9 | 2 | 0 | 3.9 |  |
| 106 | 36 |  | 4 | 9.2 | 0 | 1 |  |  |
| 107 | 36 | INTB 1a | 6.5 | 7.7 | 1 | 1 |  |  |
| 108 | 33 | INTB 1a | 5 | 6.1 | 0 | 2 |  | 4.1 |
| 109 | 37 | INTB 1a, CPx | 6 | 9.1 | 1 | 1 |  |  |
| 110 | 59 | INTB 1a | 2 | 9.2 | 1 | 1 |  |  |
| 111 | 27 | INTB 1a, CPx | 6 | 4.6 | 1 | 0 |  |  |
| 112 | 33 |  | 7 | 4.4 | 1 | 3 |  | 0.5 |
| 113 | 33 | INTB 1a | 5.5 | 9.3 | 2 | 0 | 5.8 |  |
| 114 | 52 | INTB 1a | 3.5 | 9.8 | 2 | 1 | 5.6 |  |
| 115 | 48 |  | 6 | 9.0 | 2 | 1 | 0.1 |  |
| 116 | 38 | INTB 1a | 6 | 8.9 | 1 | 1 |  |  |
| 117 | 45 | INTB 1a, CPx | 3 | 9.5 | 0 | 1 |  |  |
| 118 | 39 |  | 3.5 | 9.4 | 1 | 2 |  | 0.5 |
| 119 | 70 | INTB 1a | 6.5 | 9.0 | 3 | 2 | 0.1 | 0.6 |
| 120 | 70 | INTB 1a | 6 | 9.2 | 1 | 2 |  | 0.7 |
| 121 | 32 | INTB 1a | 5.5 | 6.9 | 0 | 1 |  |  |
| 122 | 24 |  | 2 | 9.6 | 1 | 0 |  |  |
| 123 | 28 |  | 2 | 9.2 | 0 | 1 |  |  |
| 124 | 44 | CPx | 4 | 8.4 | 0 | 2 |  | 1 |

^σ^ Previous disease modifying therapies (DMT): INTB=interferon beta, CPx=copaxone, FIN=fingolimod, TERi=teriflunomide, AZA=azathioprine.

**Supplementary code snippet 1** – Our method of bootstrapped, out-of-fold, cross-validation. The list of unique subjects is shuffled at each bootstrap iteration, and the data from each patient is added to the training dataset in 80% of the cases, and to the test dataset in the remining 20% of the cases. Cross-validation areas under the Receiver Operating Characteristic curve are stored in ROC_AUCs for later analysis.

| test_prop=0.20 # test proportion  for bootstrap_iteration in range(0,500):  shuffle(usubjects) # Shufle the list of unique subjects  for subject_index in range(0,len(usubjects)):  if subjecti<len(usubjects)*(1-test_prop):  ADD_TO_training_data()  else:  ADD_TO_test_data()  model.fit(training_data)  predictions=model.predict(test_data)  ROC_AUCs.append(ROC_AUC(test_data,predictions)) # add to the list of cross-validation ROC AUC values |
| --- |

**Supplementary code snippet 2** – Our greedy, forward-stepwise method of feature selection. The features selected are stored in SELECTED_FEATURES. The for loop determines which feature, when added to the list of SELECTED_FEATURES gathered thus far, produces the maximum area under the receiving operating characteristic curve (AUC) obtained over bootstrapped, out-of-fold, cross-validation. The process stops when no additional features that increase the AUC stored in AUC_SELECTED_FEATURES can be found.

| SELECTED_FEATURES=[]  AUC_SELECTED_FEATURES=0  while True:  MAX_AUC=0  for i in range(0,len(ALL_FEATURES)):  if ALL_FEATURES[i] in SELECTED_FEATURES:  continue  SELECTED_FEATURES.append(ALL_FEATURES[i])  AUC=eval_BSCVAUC(SELECTED_FEATURES) # evaluate AUC using bootstrapped cross-validation  if AUC>MAX_AUC:  MAX_AUC=AUC  MAX_AUC_FEATURE=ALL_FEATURES[i]  SELECTED_FEATURES= SELECTED_FEATURES[0:-1]  if MAX_AUC>AUC_SELECTED_FEATURES:  SELECTED_FEATURES.append(MAX_AUC_FEATURE)  AUC_SELECTED_FEATURES=MAX_AUC  else:  break |
| --- |
